# Synergistic Tuning of Boron-Containing Concrete for Neutron Shielding: Mix Design, Dosage Optimization, and Mechanistic Insights

**DOI:** 10.3390/ma19163404

**Published:** 2026-08-11

**Authors:** Chao Xu, Zhining Zhang, Xianglong Kong, Yi Li, Shichuan Xu, Zhihao Yang, Ye Tian

**Affiliations:** 1 Suzhou Nuclear Power Research Institute Co., Ltd., Suzhou 215004, China; xhlml881118@sina.com (C.X.); 13012205585@163.com (Z.Z.); jimmylee4801@163.com (Y.L.); 13862124726@163.com (S.X.); yzh1430653405@foxmail.com (Z.Y.); 2 Institute of Advanced Engineering Structures, Zhejiang University, Hangzhou 310058, China

**Keywords:** boron-containing radiation-shielding concrete, boron-10, mechanical properties, analytic hierarchy process (AHP), XRD analysis

## Abstract

Boron-containing concrete is an important neutron-shielding material for nuclear engineering, but the addition of boron compounds may adversely affect cement hydration and mechanical properties. This study employed boric acid and boron carbide as boron-10 neutron-absorbing sources. Within the material system investigated, the comprehensive effects of boric acid, boron carbide, and their combined use on setting time, mechanical properties, and effective boron loading were compared. MAA-based mix design and the analytic hierarchy process (AHP) were then employed to screen candidate mix proportions. Based on particle packing optimization, a series of concrete mixtures were prepared to evaluate workability, setting time, mechanical strength, boron content, and hydration products. The results show that boric acid provides good boron dispersibility but strongly retards cement hydration, leading to prolonged setting time and reduced strength. Pretreatment with calcium hydroxide and the use of an early strength admixture can partly mitigate this negative effect; the final setting time of the boric acid-containing mixtures still reached as high as 34 h 11 min. Boron carbide exhibits better chemical stability and has less influence on cement hydration. When incorporated at 5–10%, boron carbide improves the balance between mechanical performance and neutron-shielding potential. In particular, the 10% boron carbide mixture achieved a 28-day compressive strength of 47.3 MPa and a splitting tensile strength of 4.21 MPa, compared with 55.5 MPa and 6.2 MPa, respectively, for the control mixture, while maintaining relatively high strength. An analytic hierarchy process was further applied to comprehensively evaluate compressive strength, splitting tensile strength, final setting time, and boron content. Furtherly, XRD results confirmed the different interaction mechanisms of boric acid and boron carbide in cementitious systems. The study provides a framework for developing boron-containing concrete with potential neutron-shielding applications.

## 1. Introduction

As the global energy system undergoes an accelerated transition toward low-carbon, clean, and secure development, nuclear energy has re-emerged as a key component of international energy strategies owing to its high energy density, reliable baseload power supply, and exceptionally low life-cycle greenhouse gas emissions. According to statistics released by the International Atomic Energy Agency (IAEA), more than 440 nuclear power reactors have been in operation in over 30 countries worldwide as of 2023, with a combined installed capacity exceeding 400 GW [1]. Nuclear power currently accounts for approximately 9–10% of global electricity generation and supplies nearly one quarter of the world’s low-carbon electricity. In the context of intensifying climate governance pressures and growing energy security concerns, nuclear energy is increasingly recognized as a critical technological pathway for achieving deep decarbonization of power systems, improving energy supply stability, and reducing dependence on fossil fuels.

In recent years, major economies, including the United States, France, the United Kingdom, Canada, Japan, and South Korea, have successively proposed strategies to extend the service life of existing nuclear power units, develop advanced reactors, and deploy small modular reactors. Emerging economies are also accelerating nuclear power projects to meet increasing electricity demand and reduce carbon emission intensity. The International Energy Agency has emphasized that, under the global net-zero emissions scenario, nuclear installed capacity must increase substantially by the mid-21st century to support the stable operation of power systems with high renewable energy penetration [2]. Moreover, during the 28th United Nations Climate Change Conference, several countries proposed an initiative to triple global nuclear capacity by 2050, further highlighting the rising strategic importance of nuclear energy in the global low-carbon transition [3].

With the continuous expansion of nuclear energy utilization, the safe operation of nuclear facilities and radiation protection have become increasingly important. Nuclear power plants, spent fuel storage facilities, radioactive waste treatment systems, and related scientific and medical nuclear facilities generate various forms of ionizing radiation, including γ-rays, neutrons, and secondary radiation. This places stringent requirements on shielding materials in terms of radiation attenuation capacity, mechanical performance, durability, and long-term service stability. Conventional radiation-shielding materials include steel plates, lead plates, polymer composites, and concrete. Although metallic materials exhibit strong γ-ray attenuation capacity, their applications are often limited by high cost, poor construction flexibility, and environmental concerns. In contrast, concrete is widely employed as a radiation-shielding material in nuclear engineering because of its abundant raw materials, cost-effectiveness, construction convenience, high formability, and ability to simultaneously provide structural support and radiation attenuation [4]. Furthermore, the incorporation of functional components such as borides, hydrogen-rich materials, barite, hematite, and steel slag can enable the synergistic shielding of γ-rays and neutrons, thereby improving the comprehensive radiation-shielding performance of concrete [5].

Current studies on radiation-shielding concrete mainly focus on the introduction of boron-containing components, regulation of bound water content, and optimization of aggregate packing systems [6]. Among these approaches, boron has attracted considerable attention due to the excellent thermal neutron capture capability of the isotope ^10^B, whose thermal neutron absorption cross section reaches approximately 3840 barn [7]. After capturing thermal neutrons, ^10^B primarily undergoes the ^10^B (n,α)7Li reaction, producing heavy charged particles with limited penetration ability and generally avoiding the generation of high-energy secondary γ-rays. Therefore, compared with shielding designs that rely solely on high-density aggregates to enhance γ-ray attenuation, the incorporation of boron-containing materials can more effectively improve the thermal neutron absorption capacity of concrete and reduce secondary radiation risks.

Previous studies have demonstrated that even a small amount of boron-containing materials can significantly enhance the thermal neutron-shielding performance of concrete. Callan et al. [8] introduced approximately 1% boron into a concrete system and reported an increase of nearly two orders of magnitude in thermal neutron absorption capacity. This indicates that boron-containing materials can provide high shielding efficiency without substantially increasing the self-weight of the structure. Accordingly, the use of boron and its compounds offers an effective strategy for developing efficient, lightweight, and multifunctional radiation-shielding concrete [9], particularly for applications such as reactor biological shielding, spent fuel storage, radioactive waste treatment, and neutron source facilities.

Boron is commonly introduced into cement-based shielding materials through soluble boron compounds, insoluble borides, or boron-containing mineral admixtures. Among them, boric acid (H_3_BO_3_) and boron carbide (B_4_C) are two representative boron sources [10]. Boric acid can provide highly dispersed boron in cement-based systems, which is beneficial for improving thermal neutron absorption. However, as a soluble boron compound, it can participate in ionic reactions during cement hydration and significantly retard the setting and hardening processes. Previous studies have shown that boric acid and borates may delay hydration product formation, deteriorate pore structure, and adversely affect both early strength and long-term mechanical properties [11,12,13]. In contrast, boron carbide is an insoluble boride with high boron content, low density, high hardness, high-temperature resistance, and excellent chemical stability, making it a commonly used neutron absorber in nuclear shielding applications [7,14]. Because boron carbide generally exists as stable particles in cement-based matrices, its inhibitory effect on cement hydration is weaker than that of soluble boron compounds [15,16]. Appropriate incorporation of fine boron carbide particles may also improve particle packing and matrix compactness through a filling effect [17]. Nevertheless, the relatively low surface activity of boron carbide limits its interfacial bonding with cement hydration products. Excessive dosage or poor dispersion may generate weak interfacial transition zones, resulting in reduced mechanical properties. In addition, its high cost and dispersion difficulty still constrain large-scale engineering applications [18,19].

Despite substantial progress, most existing studies on boron-containing radiation-shielding concrete have focused on the optimization of single performance indicators, such as Qingjun et al. [20], who primarily investigated the quasi-static mechanical properties of UHPC incorporating boron carbide; Abdullah et al. [21], who focused on the neutron absorption performance of boron carbide concrete; and Chidiac et al. [22] and Erkoyuncu et al. [23], who reported both mechanical properties and radiation-shielding parameters. Such single-index evaluation approaches cannot fully capture the complex synergistic and competitive relationships among workability, mechanical properties, radiation-shielding capacity, and durability. Therefore, they are insufficient for the multifunctional collaborative design of radiation-shielding concrete. To address the nonlinear trade-offs among these performance indicators, a systematic multi-criteria evaluation method is required.

Due to the need to balance conflicting objectives, including mechanical properties, workability, durability, cost, and environmental impacts, multi-criteria decision-making (MCDM) methods have increasingly been applied to the evaluation and mixture-proportion optimization of concrete materials [24,25,26]. The analytic hierarchy process (AHP) determines the relative weights of evaluation criteria by constructing a hierarchical structure, conducting pairwise comparisons, and performing consistency tests [27]. The technique for order preference by similarity to ideal solution (TOPSIS) ranks alternatives according to their distances from the positive-ideal and negative-ideal solutions [28]. Ahmed et al. applied fuzzy TOPSIS to concrete mixture design to account for uncertainties in performance evaluation and decision preferences [29]. In addition to AHP and TOPSIS, compromise-ranking approaches such as VIKOR, as well as methods including ELECTRE, WASPAS, and PROMETHEE, have also been applied to construction material selection and concrete mixture optimization [24,25,30].

Nevertheless, existing MCDM studies have predominantly focused on conventional, high-performance, or green concrete, whereas their application to boron-containing radiation-shielding concrete remains limited. In particular, an integrated evaluation framework that simultaneously considers neutron-shielding potential, mechanical performance, and constructability has not been fully established. Therefore, this study employs AHP to integrate 28-day compressive strength, 28-day splitting tensile strength, final setting time, and boron content for the comprehensive optimization of boron-containing concrete mixtures.

The analytic hierarchy process (AHP) is a hierarchical decision-making method that integrates qualitative judgment and quantitative analysis [31]. By establishing a structured evaluation system, conducting pairwise comparisons, and determining the weights of different indicators, AHP enables comprehensive assessment among multiple conflicting objectives. Introducing AHP into the mix proportion optimization of radiation-shielding concrete can overcome the limitations of conventional single-index evaluation and provide a rational basis for balancing shielding efficiency, mechanical performance, and workability. However, its application in the performance evaluation and selection of nuclear-grade concrete remains limited.

In this study, boron-containing radiation-shielding concrete mixtures were designed based on the modified Andreasen and Andersen (MAA) particle packing model [32]. Specimens containing different dosages of functional components were prepared, and their workability, mechanical properties, and radiation-shielding performance were systematically tested. Subsequently, AHP was employed to comprehensively evaluate the multi-performance responses and determine the optimal dosage of functional components. Finally, X-ray diffraction (XRD) characterization was conducted on the optimized mixtures to reveal the microscopic mechanism underlying performance synergy. This study aims to provide a systematic optimization framework for the multifunctional design of radiation-shielding concrete for nuclear engineering applications, ultimately developing a concrete mixture that simultaneously satisfies workability, mechanical performance, and radiation-shielding requirements.

## 2. Materials and Experiment Setup

### 2.1. Materials and Mix Proportion of Boron-Containing Concrete

#### 2.1.1. Materials

The cement used in this study was P. O. 52.5 ordinary Portland cement produced by Shandong Shanshui Cement Group Co., Ltd. (Jinan, China). The chemical composition of cement is listed in Table 1. Class I fly ash and silica fume supplied by Henan Borun Foundry Materials Co., Ltd. (Zhengzhou, China) were used as mineral admixtures. The radiation-shielding additives consisted of high-^10^B-abundance boric acid and natural-abundance boron carbide provided by China General Nuclear Power Group (Shenzhen, China). The ^10^B-enriched boric acid had a purity of 99.9% and a ^10^B-abundance of 96%. The boron carbide contained B_4_C of 97.18% by weight and a ^10^B-abundance of 20%. The detailed chemical compositions of boric acid and boron carbide are listed in Table 2 and Table 3, respectively.

The fine aggregate was natural river sand with a fineness modulus of 2.7 and a density of 2.58 g/cm^3^. The coarse aggregate was continuously graded crushed stone with particle size ranges of 5–10 mm and 10–20 mm. The chemical admixtures included a water reducer and an early strength agent. The water reducer was an HLX standard polycarboxylate-based high-performance water reducer supplied by Shanxi Feike New Materials Technology Co., Ltd. (Yunchange, China), with a water-reducing rate of 27%. The early strength agent was provided by Qingdao Zhuonengda Construction Technology Co., Ltd. (Qingdao, China), and its main component was anhydrous calcium sulfoaluminate. In addition, to mitigate the severe retardation caused by boric acid incorporation [33], a composite pretreatment method combining calcium hydroxide pretreatment with an 8% early strength agent was employed, thereby shortening the setting time of boron-containing concrete to satisfy engineering and specification requirements.

It should be noted that all data on the constituent materials and chemical compositions presented in this chapter were obtained from test reports provided by the suppliers. In particular, the effective (^10^B) content was calculated mathematically based on the actual abundance and purity of the raw materials.

#### 2.1.2. Mix Proportions of Boron-Containing Concrete

Adding boric acid materials into concrete directly will cause a decrease in the workability of concrete and prolong the setting time. To compensate for this performance deterioration, the MAA particle packing model was adopted in this study to design boron-containing concrete. The MAA particle packing model can optimize continuous particle gradation and optimal distribution modulus, enabling multi-scale particles to gradually fill voids and minimize system porosity. Combined with the secondary pore-filling effect of hydration products, it improves material compactness, fluidity, and mechanical durability through the synergistic densification of physical packing and chemical hydration [34].

Based on the maximum particle packing theory, the MAA particle size distribution can be expressed as [35]:

(1) $$P\left(D\right)=\frac{D^{q}-D_{\min}^{q}}{D_{\max}^{q}-D_{\min}^{q}}\times 100\%$$

where P(D) is the cumulative volumetric fraction of particles with sizes less than or equal to D; D is a certain particle size; D_min_ is the minimum particle size; D_max_ is the maximum particle size; and q is the distribution modulus, which determines the shape of the gradation curve.

**Figure 1:**
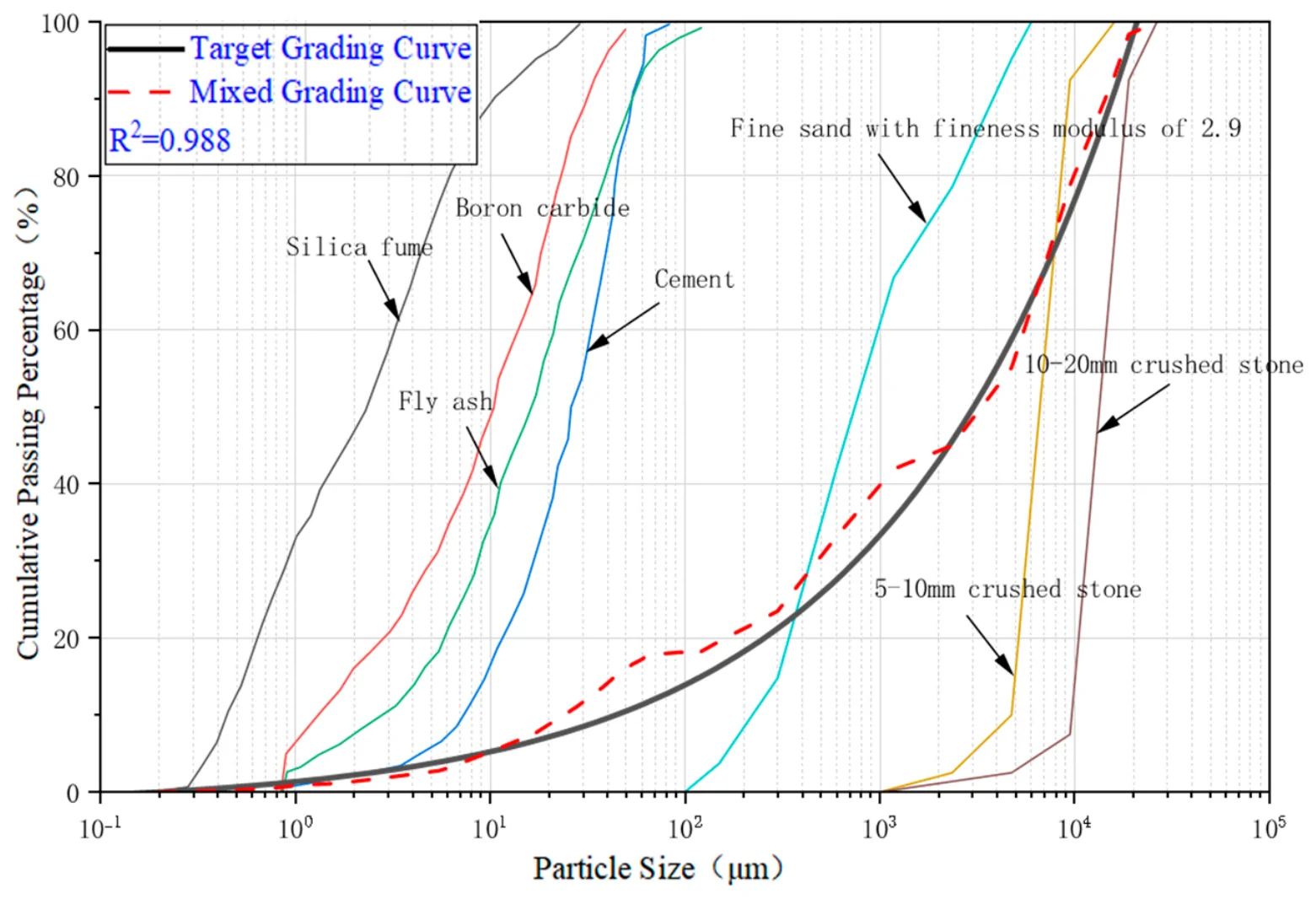
Particle size distribution of concrete raw materials.

In this study, the particle size distribution curves of silica fume, fly ash, boron carbide, cement, sand, and coarse aggregate are demonstrated in Figure 1. Based on the MAA particle packing model, the mix proportions for boron-containing concrete were designed using cement as the reference basis. Different dosages of boron-containing materials were incorporated to regulate the boron content over the range of 0–21.84‱, while a composite mineral admixture system consisting of fly ash and silica fume was employed to optimize the particle gradation and microstructure. The aggregate was designed with a continuous gradation. The sand ratio and the proportions of fine and coarse crushed stones were slightly increased with increasing boron dosage to better conform to the MAA particle packing model and achieve a higher packing density. Meanwhile, the water-reducer dosage were moderately increased, and an early strength agent was incorporated in selected mixtures to compensate for the adverse effects of boron-containing materials on workability and setting behavior. Overall, a multivariable mix proportion system characterized by progressively increasing boron content, optimized particle gradation, and admixture regulation was established to systematically investigate the effects of boron dosage on the workability and strength of concrete.

**Figure 2:**
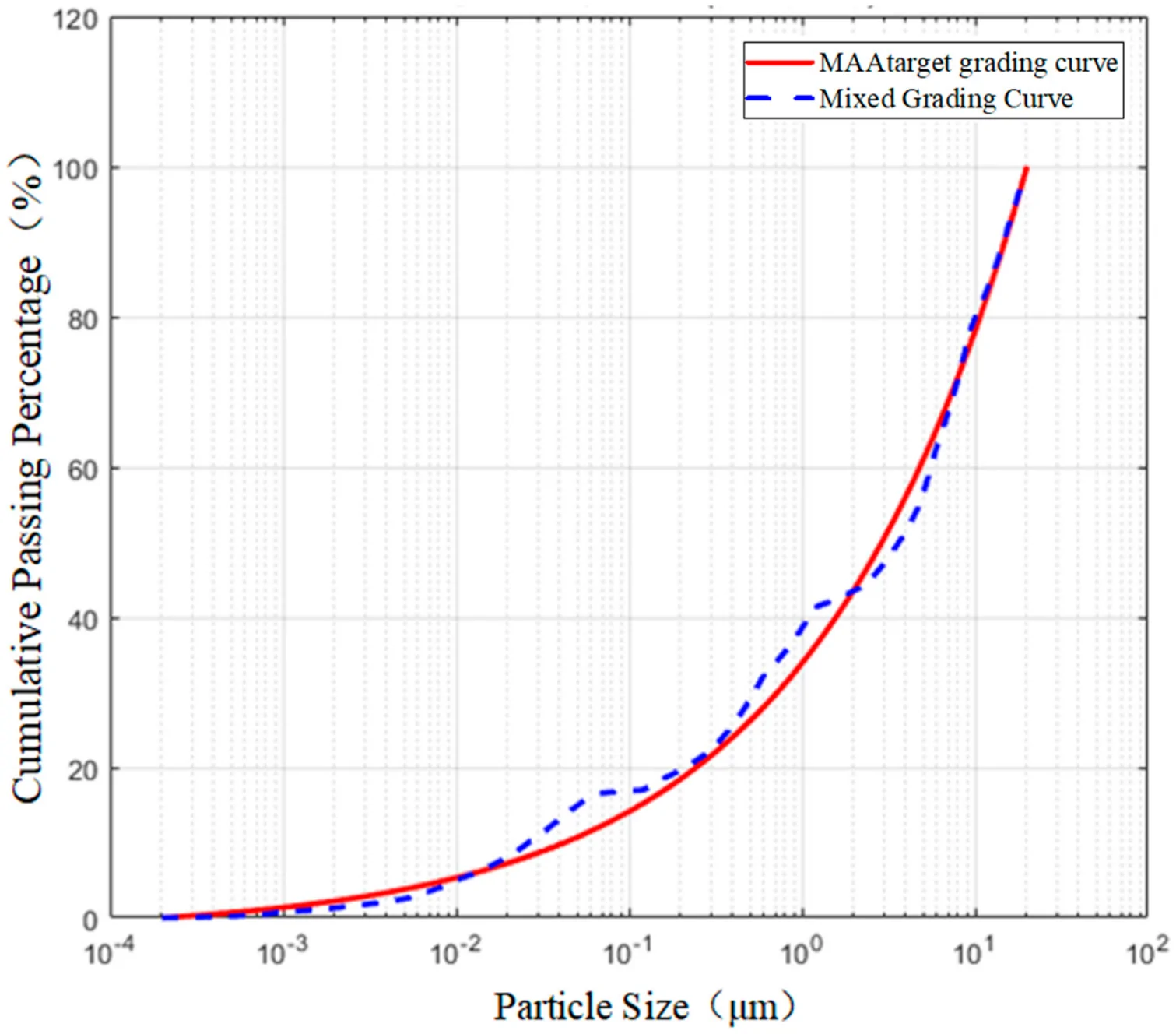
Cumulative passing percentage based on MAA model mix proportions.

As shown in Figure 2, the target cumulative passing curve of the raw materials used in boron-containing concrete is obtained. By comparing the mixed grading curve with the target grading curve, the agreement between the actual gradation and the ideal densest packing state can be evaluated. Based on this comparison, the proportions of different aggregate fractions can be optimized so that the gradation approaches the target curve, thereby improving its strength, stability, and durability. The mix proportions of boron-containing concrete are presented in Table 4. Here, KBZ concrete is the reference concrete with no boron-containing materials. For the boron-containing concrete, C denotes concrete containing boron carbide, H states concrete containing boric acid, and CH implies concrete incorporating both boron carbide and boric acid. The numbers indicate the mass percentage of the corresponding boron-containing admixture used to replace cement. In Table 4, the boron content was calculated as the mass fraction of effective ^10^B relative to the total mass of concrete.

### 2.2. Experiment Setup

#### 2.2.1. Setting Time

The setting time test was conducted in strict accordance with the Standard for Test Methods of Performance on Ordinary Fresh Concrete (GB/T 50080-2016) [36]. The setting time of boron-containing concrete was determined using the penetration resistance method. During the test, all raw materials were accurately weighed according to the predetermined mix proportions and then added into the mixer in the specified sequence, followed by thorough mixing to obtain a homogeneous fresh concrete mixture. Subsequently, the mortar fraction was separated from the fresh concrete using a 5 mm sieve, placed into the penetration resistance test container, and its surface was leveled. The specimen was then stored under controlled conditions at a temperature of 20 ± 2 °C and a relative humidity of not less than 90%.

Timing was initiated from the moment water was added for mixing. An appropriate penetration needle was selected according to the magnitude of the penetration resistance, and the needle was vertically inserted into the specimen at a uniform rate to a depth of 25 ± 2 mm within 10 ± 2 s. The penetration load and corresponding testing time were recorded. Measurements were performed every 30 min before the initial setting stage, and the interval was shortened to 15 min when approaching the initial and final setting stages, until the penetration resistance reached no less than 28 MPa. Finally, the penetration resistance was calculated based on the measured data, and the time–penetration resistance curve was plotted and fitted. The times corresponding to penetration resistance values of 3.5 MPa and 28.0 MPa were defined as the initial and final setting times, respectively. The measurement result was taken as the average of three parallel tests.

#### 2.2.2. Compressive Strength and Splitting Tensile Strength Tests

**Figure 3:**
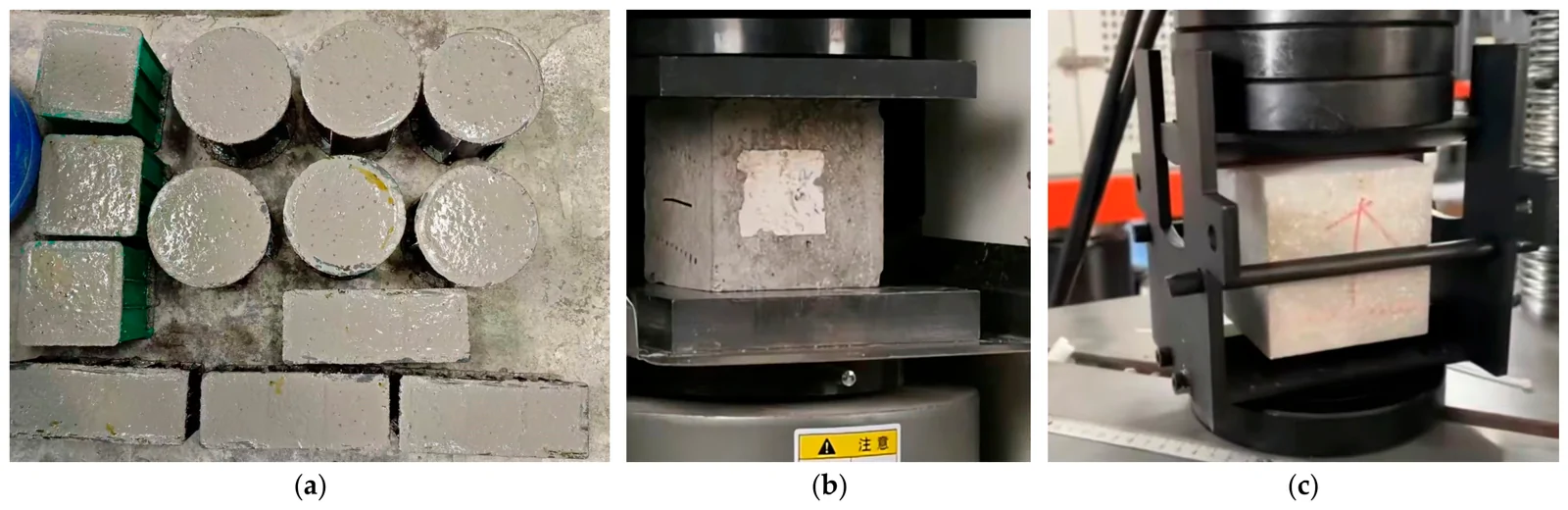
(**a**) Specimen photographs; (**b**) compressive strength testing procedure photographs; (**c**) splitting tensile strength testing procedure photographs.

The cubic compressive strength test and splitting tensile strength test of concrete were conducted following the Standard for Test Methods of Mechanical Properties of Ordinary Concrete (GB/T 50081—2019) [37]. Cubic specimens with sizes of 100 mm × 100 mm × 100 mm were prepared for the test. After casting, all specimens were cured under the standard conditions at the temperature of 20 ± 2 °C and the relative humidity of 95%. At 1 d, all the specimens were demolded and then cured under the standard conditions again until the test date. For the compressive strength test, the specimens were tested at 1 d, 3 d, 7 d, 14 d, and 28 d. For the splitting tensile strength test, the specimens were tested at 28 d. Schematic diagrams of the compressive strength test and the splitting tensile strength test are presented in Figure 3.

#### 2.2.3. XRD Analysis

For the XRD analysis, three mix proportions, namely C7.5, C10, and H4.5, were selected. The samples were collected from the cured specimens that had undergone compressive strength testing at 14 d. The obtained samples were then immersed in anhydrous ethanol for at least 24 h to terminate hydration, after which they were dried in a vacuum oven at 40 °C to a constant weight. The dried samples were ground and passed through a 300-mesh sieve to control the particle size below 45 μm. Approximately 1 g of powder was collected from each mix proportion to ensure the homogeneity and representativeness of the test samples, as well as the repeatability of the diffraction peaks. The XRD analysis was performed using a SmartLab SE diffractometer manufactured by Rigaku Corporation, Tokyo, Japan. During the test, a Cu-Kα radiation source was used, with a tube voltage of 40 kV and a tube current of 40 mA. Scanning ranges from 5° to 70°, with a step size of 0.02°/s were used to obtain high-resolution XRD patterns. The XRD data were analyzed using Jade 9 software, and phase identification was performed using the PDF-4+ 2009 crystallographic database.

#### 2.2.4. Calculation of Effective ^10^B Content

The effective ^10^B content was calculated based on the actual dosage, purity, boron mass fraction, and ^10^B isotopic abundance of each boron-containing raw material. First, the dosage of each boron-containing material per unit volume of concrete was determined from the mix proportions. The material purity was then obtained from supplier-provided test reports, while the total boron mass introduced was calculated using the theoretical or measured boron mass fraction. Subsequently, the total boron mass was multiplied by the actual isotopic abundance of ^10^B to obtain the effective ^10^B contribution of each material. Finally, the effective ^10^B contents contributed by all boron-containing materials were summed to determine the total effective ^10^B content of the concrete.

## 3. Experiment Results

### 3.1. Setting Time

Setting time is a key indicator characterizing the transition of concrete from a plastic state to a hardened state, and it directly affects the transportation, pouring, vibration, and continuous construction quality of concrete for nuclear industry building structures [38]. Soluble boron compounds can markedly retard cement hydration and consequently prolong the setting time of concrete. For boron-containing neutron-shielding concrete, it is generally recommended that the initial setting time be controlled within 3–8 h and the final setting time within 10–12 h, with a maximum final setting time of 24 h. Boron carbide is chemically stable and has a relatively limited retarding effect on cement hydration, whereas boric acid and free boron species in the system are the primary contributors to hydration inhibition and prolonged setting. Current Chinese national standards do not specify mandatory, uniform limits for the setting time of boron-containing neutron-shielding concrete; relevant control criteria are generally determined according to material characteristics, construction scheduling, and practical requirements for placing, compaction, and coordination between construction procedures.

**Figure 4:**
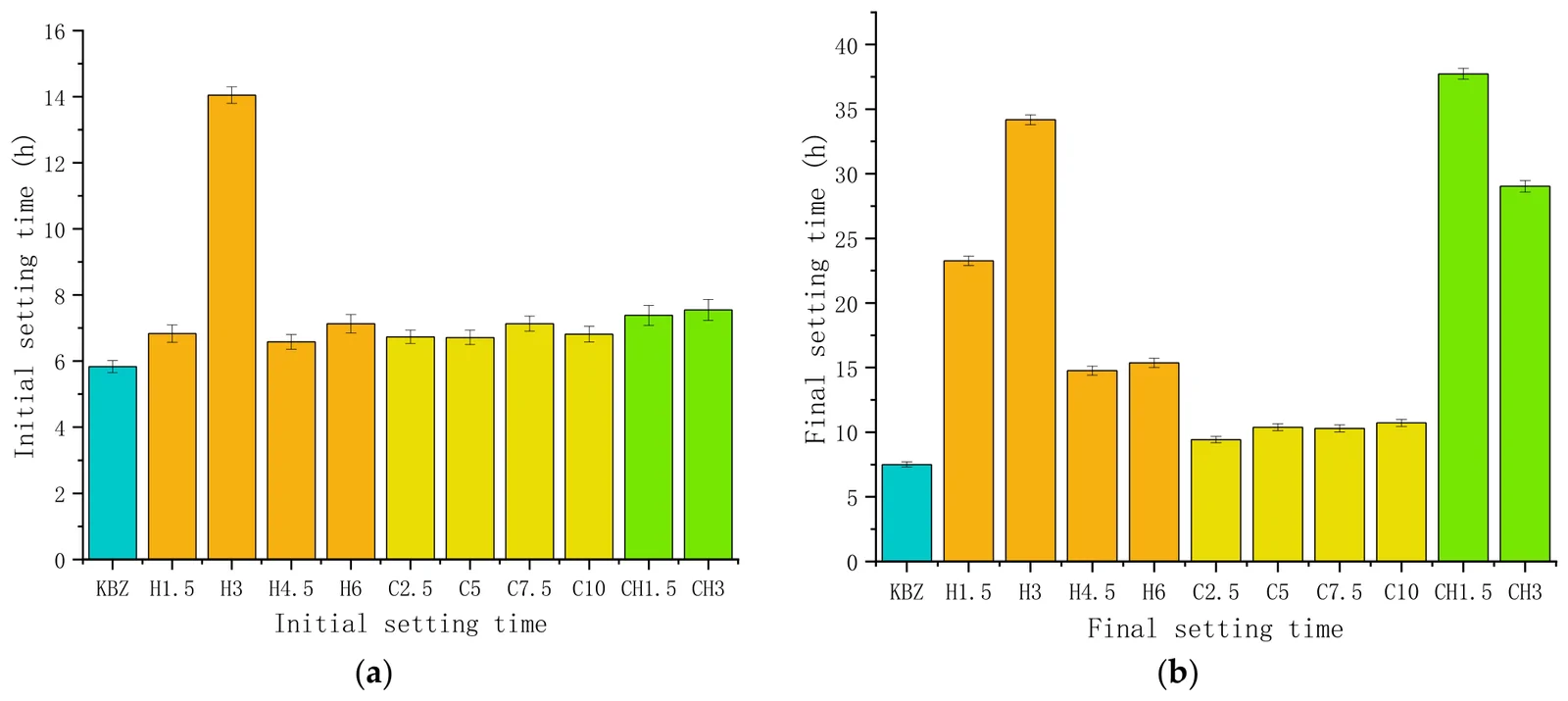
Setting time of different concretes: (**a**) initial setting time; (**b**) final setting time.

Existing studies have shown that boron-containing materials inhibit cement hydration reactions, resulting in prolonged setting time and possibly affecting early strength development [39]. In radiation-shielding concrete, radiation-shielding constituents such as boric acid and boron carbide, mineral admixtures such as fly ash and silica fume, and chemical admixtures such as water-reducing and early strength agents are incorporated simultaneously and may exert coupled effects on the properties of the concrete. Therefore, it is necessary to investigate the variation in the setting time of boron-containing concrete, so as to conduct a comparative evaluation and selection of mix proportions. And it is reasonable to find out that boric acid and boron carbide exhibit distinctly different influence on the setting behavior of concrete, as shown in Figure 4.

The sole incorporation of boric acid markedly extends both the initial and final setting times of concrete, suggesting that boric acid strongly inhibits early-stage cement hydration. Among all mixtures, the H3 group exhibits the most pronounced retarding effect, with the initial and final setting times increasing by 141% and 356%, respectively, compared with the reference concrete. This indicates that boric acid at a dosage of 3% exerts the strongest inhibitory effect on the cement hydration process. Notably, the increase in final setting time is much greater than that in initial setting time, implying that boric acid not only delays the loss of plasticity of the cement paste but also more substantially suppresses the continuous formation of hydration products and the development of the hardened structure at later stages. Previous studies suggest that, after entering the cementitious system, boric acid or borate delays cement hydration by modifying the pore solution environment, affecting calcium ion dissolution and the precipitation of hydration products, and potentially forming hydrated calcium borate phases [40]. When the boric acid dosage further increases to 4.5% and 6%, the average increase in initial setting time decreases to 17.6%, whereas the average increase in final setting time remains as high as 101%. This indicates that the effects of boric acid on the initial and final setting stages are not synchronized. Compared with the H3 group, the setting times of the high-dosage groups do not continue to increase but instead decrease to some extent, suggesting that the relationship between boric acid dosage and setting time is not simply linear. Based on the existing literature, it can be inferred that, under high-dosage boric acid conditions, the reaction between borate and calcium ions, the formation of calcium borate products, and the interaction with chemical admixtures jointly modify the reaction environment on the surface of cement particles, thereby leading to a nonlinear variation in setting time characterized by an initial sharp prolongation followed by a decline and subsequent stabilization [41]. From the perspective of construction adaptability, the setting time of the H4.5 group is relatively appropriate. It maintains a certain retarding effect while avoiding the excessively long construction waiting time and delayed early strength development associated with the excessive retardation observed in the H3 group.

In contrast, the sole incorporation of boron carbide exerts a much weaker influence on the setting time. In the test, the initial setting time of the boron carbide-containing mixtures increases from 5 h 50 min for the blank group to a maximum of 7 h 8 min, corresponding to an increase of 15.1–22.3%. Meanwhile, the final setting time increases from 7 h 30 min to a maximum of 10 h 43 min, with an increase of 25.8–42.9%. These results indicate that, although boron carbide delays the setting and hardening of concrete to some extent, its retarding effect is substantially weaker than that of boric acid and remains relatively mild and controllable. This behavior may be attributed to the high chemical stability of boron carbide and its limited direct participation in hydration reactions under the alkaline environment of cementitious systems. Therefore, compared with boric acid, boron carbide is more suitable as a boron-containing radiation-shielding component because it causes less disturbance to the setting behavior of concrete.

Under comparable total boron contents, the setting time of the mixtures incorporating both boric acid and boron carbide is significantly longer than that of the corresponding mixtures with either component alone. This suggests that the two boron-containing components exert a combined inhibitory effect on the cement hydration process. Specifically, boric acid can rapidly dissolve into the pore solution during the early mixing stage and suppress early cement hydration, whereas boron carbide may induce continuous interfacial blocking or slow-release effects in the system owing to its relatively stable particulate characteristics. The combined action of these two components therefore results in more pronounced retardation in the composite system than in the single-incorporation systems.

### 3.2. Compressive Strength

**Figure 5:**
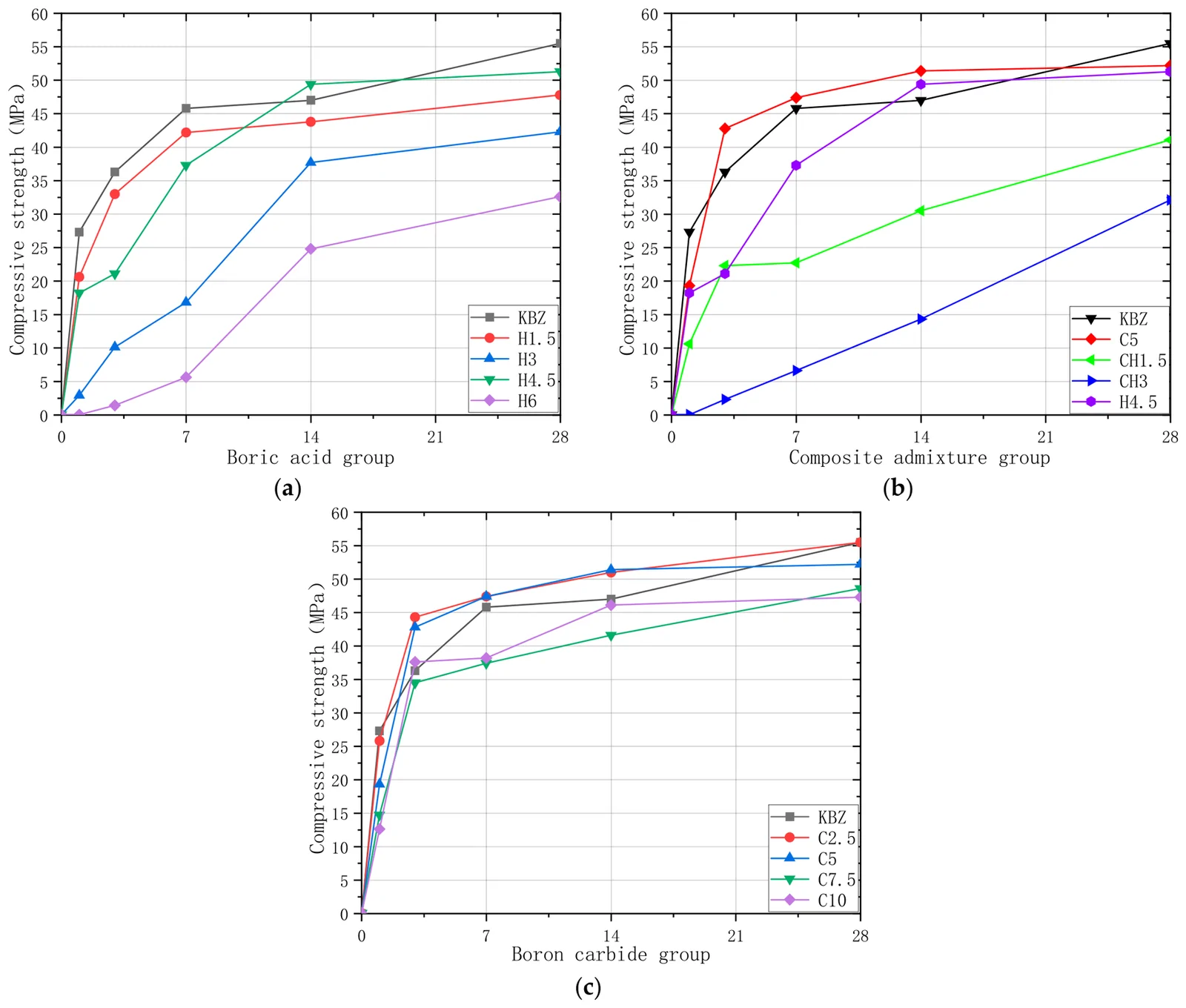
Development of compressive strength with age: (**a**) boric acid group; (**b**) boron carbide group; (**c**) composite admixture group.

The cubic compressive strength results of boron-containing concrete are presented in Figure 5. As shown in Figure 5, the development of compressive strength for boric acid concrete exhibits a characteristic trend of early-stage inhibition followed by rapid later-stage recovery. At curing ages of 1–3 d, the retarding effect of boric acid remains dominant, leading to a pronounced suppression of early cement hydration. In particular, the 1 d compressive strength of the high-dosage group with 6% boric acid is nearly zero, indicating that excessive boric acid severely delays the formation of the early hardened structure. In contrast, the early strengths of the 1.5% and 4.5% dosage groups are improved, suggesting that the adverse effects of low- and medium-dosage boric acid on hydration can be alleviated to some extent when an appropriate boric acid content is used in combination with composite modification measures.

After 7 d, the compressive strength of all concrete groups undergoes a rapid growth, indicating that the hydration reactions inhibited at early ages gradually recover and proceed at later ages. Among these mixtures, the H4.5 group achieves the highest 28 d compressive strength of 51.3 MPa, demonstrating the most favorable mechanical performance. By contrast, the 28 d compressive strength of the high-dosage group with 6% boric acid is only 32.6 MPa, indicating that excessive boric acid still markedly impairs the formation and densification of the internal concrete structure, thereby limiting later-age strength development. Overall, an appropriate boric acid dosage is beneficial for balancing later-age strength growth and structural stability, whereas an excessive dosage leads to significant deterioration in the mechanical properties of concrete.

The influence of boron carbide on the compressive strength of concrete exhibits pronounced age dependence and dosage sensitivity. At the early age of 1 d, the compressive strength continuously decreases with increasing boron carbide dosage. The 1 d compressive strength of the reference group is 27.3 MPa, whereas those of the 2.5%, 5%, 7.5%, and 10% dosage groups decrease to 25.8, 19.3, 14.7, and 12.6 MPa, respectively. This indicates that the incorporation of boron carbide inhibits the early strength development of concrete. This behavior is mainly attributed to the low reactivity of boron carbide particles. On the one hand, boron carbide reduces the effective content of cementitious materials per unit volume, thereby producing a dilution effect. On the other hand, the adsorption effect on the particle surface may affect ion migration and the formation of hydration products around cement particles, consequently delaying the development of the early hardened structure. With increasing curing age, the strength of the low-dosage boron carbide groups gradually recovers and exhibits a certain enhancement effect at the intermediate age.

At 14 d, the compressive strengths of the 2.5% and 5% dosage groups reach 51.0 MPa and 51.4 MPa, respectively, both of which are higher than that of the blank group, 47.0 MPa. This suggests that the improved intermediate-age strength at an appropriate boron carbide dosage may be associated with a potential particle-filling effect. However, its effects on the pore structure and interfacial transition zone were not directly examined in this study and require further verification by pore structure characterization and SEM/EDS observations. At 28 d, the effect of boron carbide on compressive strength shows a threshold-dependent characteristic. The compressive strength of the 2.5% dosage group is 55.5 MPa, which is nearly comparable to that of the blank group, indicating that low-dosage boron carbide has only a limited effect on later-age strength. In contrast, the strengths of the 7.5% and 10% dosage groups decrease to 48.6 MPa and 47.3 MPa, respectively, indicating that excessive boron carbide weakens the later-age mechanical properties of concrete. This reduction may be associated with the dilution effect caused by excessive boron carbide, which decreases the effective cementitious-material content. Possible particle agglomeration and changes in the interfacial region may also contribute.

In summary, the effect of boron carbide on concrete strength is not simply inhibitory but is jointly governed by dosage and curing age. Appropriate incorporation improves intermediate-age strength through the filling effect, whereas excessive dosage results in strength deterioration due to the dominant dilution effect and the formation of interfacial defects.

In addition, the test results show that the combined incorporation of boric acid and boron carbide leads to significantly lower compressive strength at all curing ages than the single incorporation of either boric acid or boron carbide. This indicates that the retarding effect of boric acid and the dilution effect of boron carbide on cementitious materials may coexist in the composite system, resulting in more pronounced inhibition of early cement hydration. The lower strength of the combined incorporation mixtures may be associated with the concurrent retarding effect of boric acid and the dilution effect of boron carbide. These factors may delay early hydration and reduce the development rate of the cementitious matrix. In addition, changes in particle dispersion and interfacial bonding may also contribute to the observed strength reduction. Since SEM/EDS observations, pore structure characterization, and quantitative phase analysis were not conducted, the effects on hydration products, pore structure, and the interfacial transition zone should be regarded as possible explanations rather than confirmed mechanisms.

### 3.3. Splitting Tensile Strength

**Figure 6:**
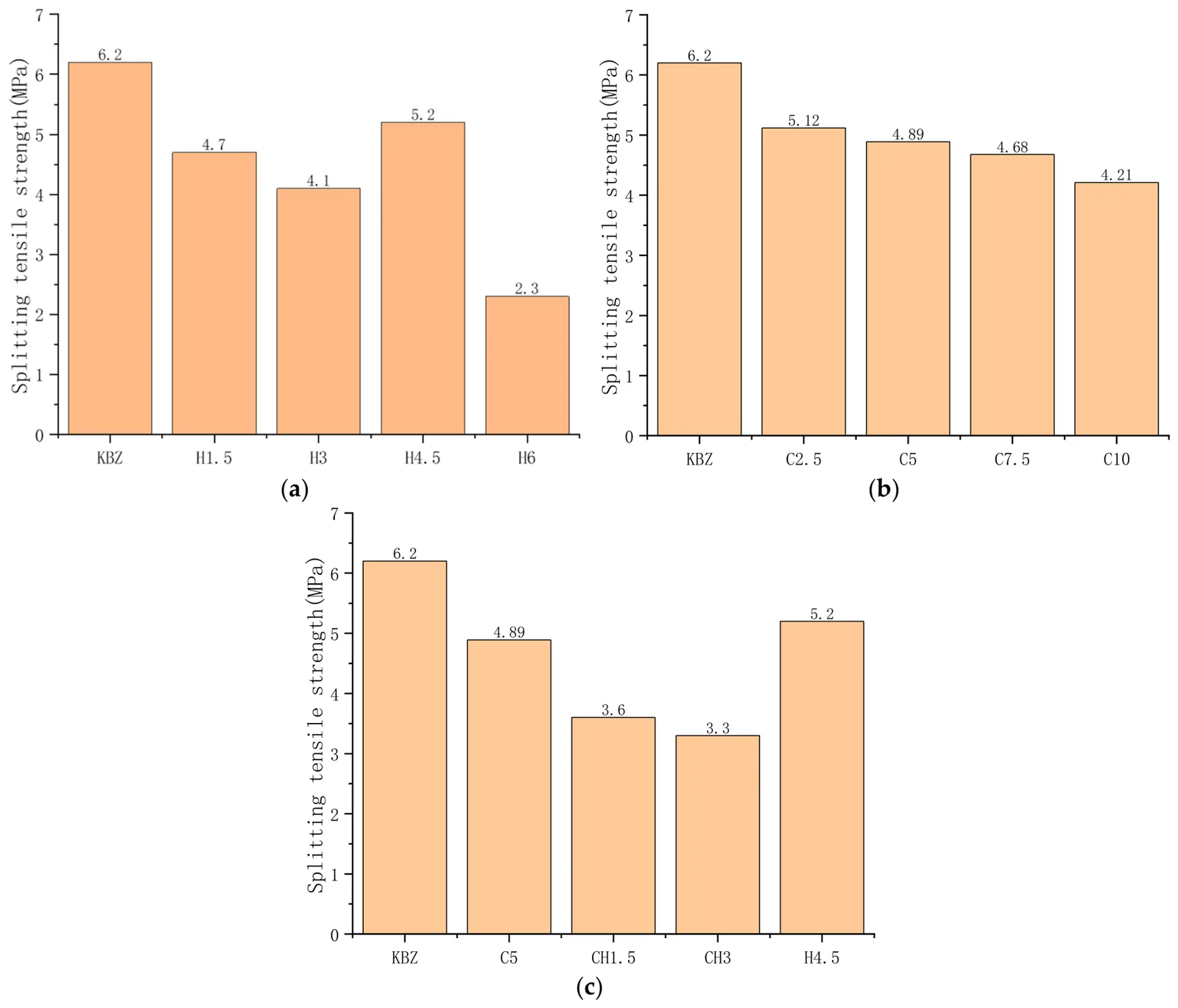
Development splitting tensile strength with time: (**a**) boric acid group; (**b**) boron carbide group; (**c**) composite admixture group.

The splitting tensile strength results of the boron-containing concrete are presented in Figure 6. As shown in Figure 5, the splitting tensile behavior of boric acid concrete exhibits a markedly different evolution pattern from that of the boron carbide mixtures. With increasing boric acid dosage, the splitting tensile strength does not decrease linearly but instead shows a nonlinear fluctuation. Specifically, the splitting tensile strengths of the 1.5% and 3% boric acid groups are 4.7 and 4.1 MPa, respectively, corresponding to reductions of 24.2% and 33.9% compared with the blank group. Notably, when the boric acid dosage increases to 4.5%, the splitting tensile strength rebounds to 5.2 MPa, which is significantly higher than those of the 1.5% and 3% groups. This atypical strength recovery suggests that the pretreatment process may exert a synergistic strengthening effect at an appropriate boric acid concentration. At a dosage of 4.5%, the calcium borate particles generated by CH pretreatment and the hydration products promoted by the early strength agent may achieve a relatively dense spatial distribution, thereby forming a more effective micro-aggregate filling system. This physical filling effect partially offsets the adverse influence of borate ions on the bonding capacity of cementitious materials and optimizes stress distribution under tensile loading, consequently improving the load-bearing capacity of the material before brittle fracture. However, when the boric acid dosage reaches 6%, the compensatory effect of the modification process becomes limited, and the splitting tensile strength decreases sharply to 2.3 MPa, corresponding to a reduction of approximately 62.9% compared with the blank group.

The splitting tensile strength of concrete exhibits a monotonically decreasing trend with increasing boron carbide dosage. The splitting tensile strength of the blank control group is 6.20 MPa, indicating that the reference concrete possesses favorable matrix bonding capacity. When the boron carbide dosage is 2.5%, the splitting tensile strength decreases to 5.12 MPa, representing a reduction of 17.4% compared with the blank group. As the dosage further increases to 5%, 7.5%, and 10%, the splitting tensile strengths decrease to 4.89, 4.68, and 4.21 MPa, respectively, with progressively greater reductions. These results indicate that the incorporation of boron carbide adversely affects the tensile performance of concrete, and this detrimental effect becomes more pronounced with increasing dosage. This behavior may be attributed to the low reactivity of boron carbide particles, which tend to form local agglomerations within the matrix when incorporated at excessive dosages, thereby weakening the continuity of the hardened cement paste structure. In addition, the interfacial bonding between boron carbide particles and the cement matrix is relatively weak, which facilitates the formation of weak zones for microcrack initiation and propagation, ultimately resulting in a reduction in the splitting tensile strength of concrete [9].

In the design logic of composite shielding materials, the data of the mixed groups show that the 28 d splitting tensile strength of the blank control group is 6.2 MPa, while the strengths of the CH1.5 group and the CH3 group are measured as 3.6 MPa and 3.3 MPa, respectively. Under the premise that the total dosage is approximately 4.5%, the tensile strengths of the two mixed specimens decrease by 41.9% and 46.8%, respectively, compared with the blank group. Moreover, the tensile performance of the mixed groups of boric acid and boron carbide is significantly lower than that of the single incorporation boric acid and boron carbide groups.

## 4. Comparative Evaluation and Selection of Boron-Containing Concrete Based on AHP Method

### 4.1. Application of AHP Method

In this study, the AHP method is adopted to evaluate and optimize the comprehensive performance of boron-containing radiation-shielding concrete with different mix proportions. The essence of AHP lies in decomposing complex multi-criteria decision-making problems into a hierarchical structure. By establishing a systematic framework consisting of the goal layer, criterion layer, and alternative layer, AHP enables a quantitative assessment of the relative contribution of each performance indicator to the final optimal selection. In accordance with the objectives of this study, the goal layer is defined as the optimal selection of the comprehensive performance of boron-containing radiation-shielding concrete. The criterion layer includes four indicators, namely compressive strength, splitting tensile strength, boron content, and setting time, which are used to characterize the load-bearing capacity, crack resistance, neutron-shielding potential, and construction workability of concrete, respectively. The alternative layer comprises the experimental groups containing boric acid, boron carbide, and their combined incorporation, which are further evaluated and ranked to determine the optimal mix proportion.

In the process of weight determination, a pairwise comparison judgment matrix is firstly established based on the service requirements of boron-containing radiation-shielding concrete and the relative importance of each performance indicator. The scale method is employed to quantify the importance of the criterion layer indicators with respect to the target layer, where a value of 1 indicates equal importance between two indicators, whereas a value of 9 represents the extreme importance of one indicator over another. For boron-containing radiation-shielding concrete, boron content directly affects neutron absorption capacity and thus serves as a critical parameter for evaluating shielding performance. Compressive strength and splitting tensile strength are associated with the load-bearing safety and crack resistance durability of structural components, respectively. Setting time reflects the construction adaptability of the material. Therefore, the constructed judgment matrix can comprehensively characterize the relative contribution of each indicator in radiation-shielding structural applications.

From a mathematical perspective, AHP determines the weight coefficients of compressive strength, splitting tensile strength, boron content, and setting time in the comprehensive evaluation by calculating the maximum eigenvalue of the judgment matrix and its corresponding eigenvector, followed by eigenvector normalization. To ensure the rationality of the weight allocation, a consistency test is performed on the judgment matrix. Specifically, the logical consistency of the matrix is evaluated by calculating the consistency ratio (CR), which is defined as the ratio of the consistency index (CI) to the random consistency index (RI). In general, when CR < 0.1, the judgment matrix is considered to exhibit acceptable consistency, indicating that the pairwise comparisons satisfy the conventional AHP consistency requirement. When CR ≥ 0.1, the pairwise comparison values should be adjusted until the consistency requirement is satisfied [42]. Through the above procedure, AHP transforms the comprehensive trade-off among shielding performance, mechanical performance, and construction performance of boron-containing radiation-shielding concrete into quantitative ranking results, thereby providing a theoretical basis for ranking alternative mix proportions under the selected evaluation criteria and weights.

### 4.2. Hierarchical Structure Calculation

**Figure 7:**
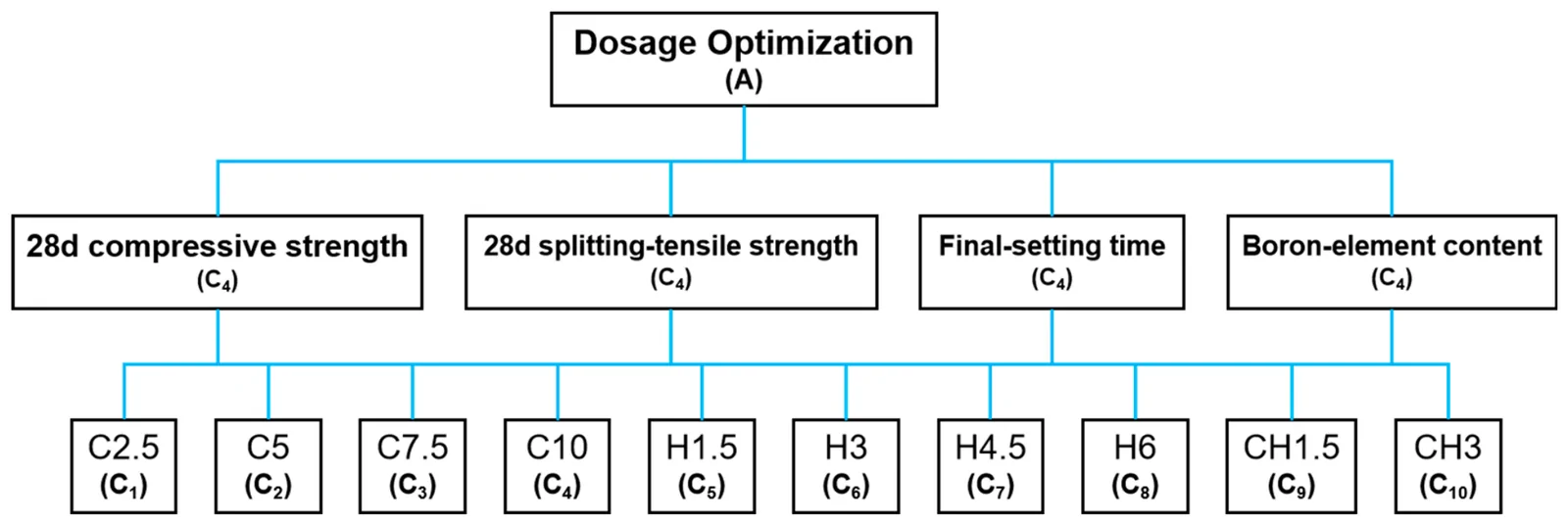
Hierarchical structure model for performance evaluation.

For boron containing radiation-shielding concrete, performance evaluation is more complex than that of ordinary concrete because it requires a comprehensive balance among structural load-bearing capacity, crack resistance, and neutron-shielding efficiency. The evaluation system established in this study aims to optimize the mix proportion of boron-containing concrete with both high mechanical strength and enhanced shielding performance. Accordingly, 28 d compressive strength, 28 d splitting tensile strength, boron content, and final setting time were selected as the evaluation criteria, forming the hierarchical structure model shown in Figure 7.

In this model, the goal layer (A) is defined as the comprehensive performance optimization of boron-containing radiation-shielding concrete. The criterion layer (B) consists of 28 d compressive strength (B1), 28 d splitting tensile strength (B2), final setting time (B3), and boron content (B4). The alternative layer (C) includes different mix proportion schemes, including single-incorporation boron carbide groups, single-incorporation boric acid groups, and boron carbide–boric acid hybrid groups. This hierarchical model provides a structural basis for the subsequent construction of the judgment matrix and weight calculation using YAAHP software V2.8.

#### 4.2.1. Judgment Matrix

In the comprehensive evaluation model, the contributions of different performance criteria to the overall objective vary significantly. Therefore, it is necessary to establish a judgment matrix for the criterion layer and to quantify the relative weights of these criteria with respect to the overall objective through pairwise comparisons of their importance. In this study, the elements of the judgment matrix were defined strictly according to the 1–9 scale and reciprocal scale method proposed by Saaty [42]. To ensure the scientific validity and engineering applicability of the derived weights, ten senior experts and engineers from nuclear power design institutes, neutron physics research institutions, and nuclear power construction units were invited to participate in the evaluation. Considering the dual requirements of structural safety and radiation-shielding functionality in nuclear power engineering practice, the experts assessed the relative importance of four criteria: compressive strength, splitting tensile strength, final setting time, and boron content. After expert scoring and subsequent averaging, the judgment matrix for the criterion layer was established, as presented in Table 5. Detailed information on the 10 experts involved in the AHP criteria-level assessment is presented in Table 6.

From the logical allocation of the criterion layer judgment matrix, the compressive strength (B1) at 28 d is assigned the highest weight. This is primarily because nuclear power protective structures, as load-bearing components, must first satisfy the fundamental requirement of structural safety under extreme service conditions. The main objective of this study is to maximize the boron content while ensuring that the basic workability and mechanical properties of concrete are not significantly degraded. As boron content (B4) represents the key functional indicator of radiation-shielding concrete, its importance is ranked second only to compressive strength. In addition, borate ions exert a pronounced inhibitory effect on cement hydration; therefore, construction feasibility is regarded as a critical consideration in the proposed evaluation system. Accordingly, the weight of final setting time (B3) is prioritized over that of 28 d splitting tensile strength (B2). This weighting scheme reflects the design philosophy of boron-containing radiation-shielding concrete, namely, structural safety as the foundation, shielding performance as the priority, and construction feasibility as the orientation. On the premise of ensuring sufficient load-bearing capacity and adequate hardening efficiency of the cementitious matrix to meet construction-cycle requirements, the neutron absorption performance of the material is maximized, while its crack resistance capacity is also taken into consideration.

For the determination of scheme-level weights, this study did not rely merely on subjective expert judgment. Instead, it incorporated the measured performance data of each radiation-shielding concrete mixture to enable quantitative evaluation. Specifically, for benefit-type indicators, such as compressive strength, splitting tensile strength, and boron content, the measured experimental results were used as the basis for constructing judgment matrices. Pairwise ratios among different schemes were calculated to characterize the relative advantages of each mix proportion with respect to the corresponding performance indicator. For instance, the concrete mixtures containing only boron carbide, only boric acid, and combined boric acid–boron carbide exhibited differences in mechanical properties and boron incorporation efficiency. Therefore, using ratios derived from measured values can more directly quantify the influence of different dosage combinations on compressive strength, splitting tensile strength, and potential radiation-shielding performance.

For the final setting time indicator, which reflects the setting and hardening process of concrete as well as its construction operability, a cost-type attribute is assigned, as lower values indicate better performance. If the measured setting time is directly used for comparison, mixtures with longer final setting times will achieve higher weights in the judgment matrix, which would be inconsistent with practical engineering requirements. Therefore, in this study, the reciprocal method was adopted to positively transform the final setting time. Specifically, the reciprocal of the pairwise ratio of measured values was used to construct the judgment matrix, ensuring that mixtures with shorter final setting times and higher construction efficiency were assigned higher priority in the evaluation system. This treatment reasonably converts the adverse effect of prolonged setting time on construction performance into a positive evaluation outcome and prevents deviations in the comprehensive ranking caused by differences in indicator attributes.

Compared with the traditional 1–9 scale based on linguistic judgments, constructing the scheme layer judgment matrix using ratios derived from measured data can more accurately reflect the actual influence of different dosages of boron-containing components on concrete performance in this study. The influences of boron carbide, boric acid, and their combined incorporation on mechanical strength, boron content, and setting time are not fully consistent; therefore, subtle differences among the schemes are difficult to distinguish accurately using subjective judgment alone. By adopting the ratio-based method, experimental results, including variations in compressive strength, reductions in splitting tensile strength, increases in boron content, and prolongation of setting time, can be converted into judgment matrices within the AHP framework. Consequently, the scheme ranking is established on the basis of measured data, which enhances the transparency and interpretability of the evaluation process.

Based on the above principles, this study constructed scheme layer judgment matrices for the four criteria, namely compressive strength, splitting tensile strength, boron content, and setting time. Detailed judgment matrices for the alternative schemes under each criterion are presented in Table 7, Table 8, Table 9 and Table 10.

#### 4.2.2. Hierarchical Single Ranking

Hierarchical single ranking is a core computational procedure in the analytic hierarchy process (AHP). Its purpose is to determine the relative importance weights of factors at a given level with respect to a specific criterion or objective at the upper level. In the comprehensive evaluation of radiation-shielding concrete, the criterion-level weights are determined based on expert judgments, while the scheme-level evaluation is based on experimentally measured performance data. To ensure a clear and robust calculation process, the arithmetic mean method (also known as the sum method) was adopted to determine the weight vector. The main steps are as follows.

(1) Column normalization of the judgment matrix

Let the judgment matrix be $A={\left(a_{\mathrm{ij}}\right)}_{n\times n}$. Each element is normalized by the sum of the corresponding column to obtain the normalized matrix $B={\left(b_{\mathrm{ij}}\right)}_{n\times n}$:

$$b_{\mathrm{ij}}=\frac{a_{\mathrm{ij}}}{\sum_{i=1}^{n}a_{\mathrm{ij}}}$$

where $a_{\mathrm{ij}}$ represents the relative importance of factor i compared with factor j.

(2) Row summation

The elements in each row of the normalized matrix are summed to obtain the row-sum vector:

$$s_{i}=\overset{n}{\underset{j=1}{\sum }}b_{\mathrm{ij}}$$

where $s_{i}$ denotes the sum of the normalized values in the i-th row.

(3) Normalization of the weight vector

The row-sum vector is further normalized to obtain the weight vector $W={\left(w_{1},w_{2},\cdots ,w_{n}\right)}^{T}$:

$$w_{i}=\frac{S_{i}}{\sum_{i=1}^{n}S_{i}}$$

where $w_{i}$ represents the relative weight of factor i.

(4) Calculation of the maximum eigenvalue

Based on the calculated weight vector, the maximum eigenvalue of the judgment matrix is calculated as the basis for the subsequent consistency test:

$$\lambda_{\max}=\frac{1}{n}\overset{n}{\underset{i=1}{\sum }}\frac{{\left(\mathrm{AW}\right)}_{i}}{w_{i}}$$

where ${\left(\mathrm{AW}\right)}_{i}$ is the i-th component of the product of matrix A and weight vector W.

Using the above procedure, the weights of the criteria, including compressive strength, splitting tensile strength, final setting time, and boron content, relative to the overall evaluation objective can be obtained. In addition, the priority rankings of different concrete mixtures under each criterion can be determined using the measured experimental data.

It should be noted that the criterion-level judgment matrix was established through pairwise comparisons by experts, whereas the scheme-level evaluation was calculated from normalized experimental results. This approach combines engineering knowledge with objective test data, enabling the conversion of multiple performance indicators into quantitative weights. All judgment matrices were subjected to consistency testing to ensure logical coherence and to reduce potential bias arising from subjective judgments.

#### 4.2.3. Hierarchical Total Ranking

After completing the single ranking at each level, hierarchical total ranking is conducted to synthesize the priorities of all alternative schemes relative to the overall objective. The total ranking represents the cumulative propagation of weights from the criterion level to the scheme level and provides the final basis for mixture optimization.

Let $W={\left(w_{1},w_{2},\cdots ,w_{n}\right)}^{T}$ denote the weight vector of the criteria relative to the target layer, and let $P_{k}$ represent the single-ranking weight vector of the alternative schemes under criterion k. The composite weight vector P of the scheme layer relative to the overall objective is calculated as:

$$P=\overset{n}{\underset{k=1}{\sum }}w_{k}p_{k}$$

where $w_{k}$ is the weight of criterion k, and P represents the composite priority vector of all schemes relative to the overall objective.

Through this weighted synthesis, the discrete performance indicators—namely compressive strength, splitting tensile strength, final setting time, and boron content—are integrated into a unified comprehensive evaluation index. The resulting total ranking reflects not only the performance of each mixture under individual mechanical or functional indicators, but also its overall balance between structural performance, workability, and potential neutron-shielding capability.

Accordingly, the total ranking makes it possible to identify mixture proportions that may not be optimal for a single indicator but exhibit the most favorable overall performance. This provides a quantitative basis for selecting boron-containing concrete mixtures with coordinated mechanical properties and neutron-shielding potential for nuclear engineering applications.

#### 4.2.4. Consistency Test

In addition, as the judgment matrix may be influenced by subjective cognition and the complexity of trade-offs among different indicators, a consistency test is required to determine whether obvious logical contradictions existed in the pairwise comparison results. When the judgment matrix satisfies the consistency requirements, the resulting weight allocation is considered to have acceptable logical rationality. Conversely, if the consistency requirement is not satisfied, the judgment matrix needs to be adjusted accordingly. Through the consistency test, the internal logical consistency of the pairwise comparison matrices can be assessed.

The judgment logic for consistency verification is based on the degree of deviation between the maximum eigenvalue $\lambda_{\max}$ of the judgment matrix and the matrix order n. According to matrix theory, when a pairwise comparison matrix reaches a state of complete consistency, its maximum eigenvalue should be exactly equal to the matrix order. However, in the actual evaluation of the comprehensive performance of boron-containing radiation-shielding concrete, due to the need to balance the nonlinear relationship between mechanical-bearing indicators, such as compressive strength and splitting tensile strength, and functional indicators, such as boron content, $\lambda_{\max}$ is usually slightly greater than n. To quantify this deviation, the consistency index CI is introduced and calculated as follows:

(2) $$\mathrm{CI}=\frac{\lambda_{\max}-n}{n-1}$$

Here, CI reflects the degree to which the judgment matrix deviates from complete consistency. That is, the closer the CI value is to 0, the better the logical consistency of the judgment matrix. To objectively measure the validity of the CIvalue, it must be compared with the random consistency index RI of the same order. The standard value of RI depends on the matrix order n, and the corresponding relationship is shown in Table 11. The parameters, equations, and numerical values involved in the analytic hierarchy process (AHP) are presented in Table 12.

#### 4.2.5. Evaluation Results

**Figure 8:**
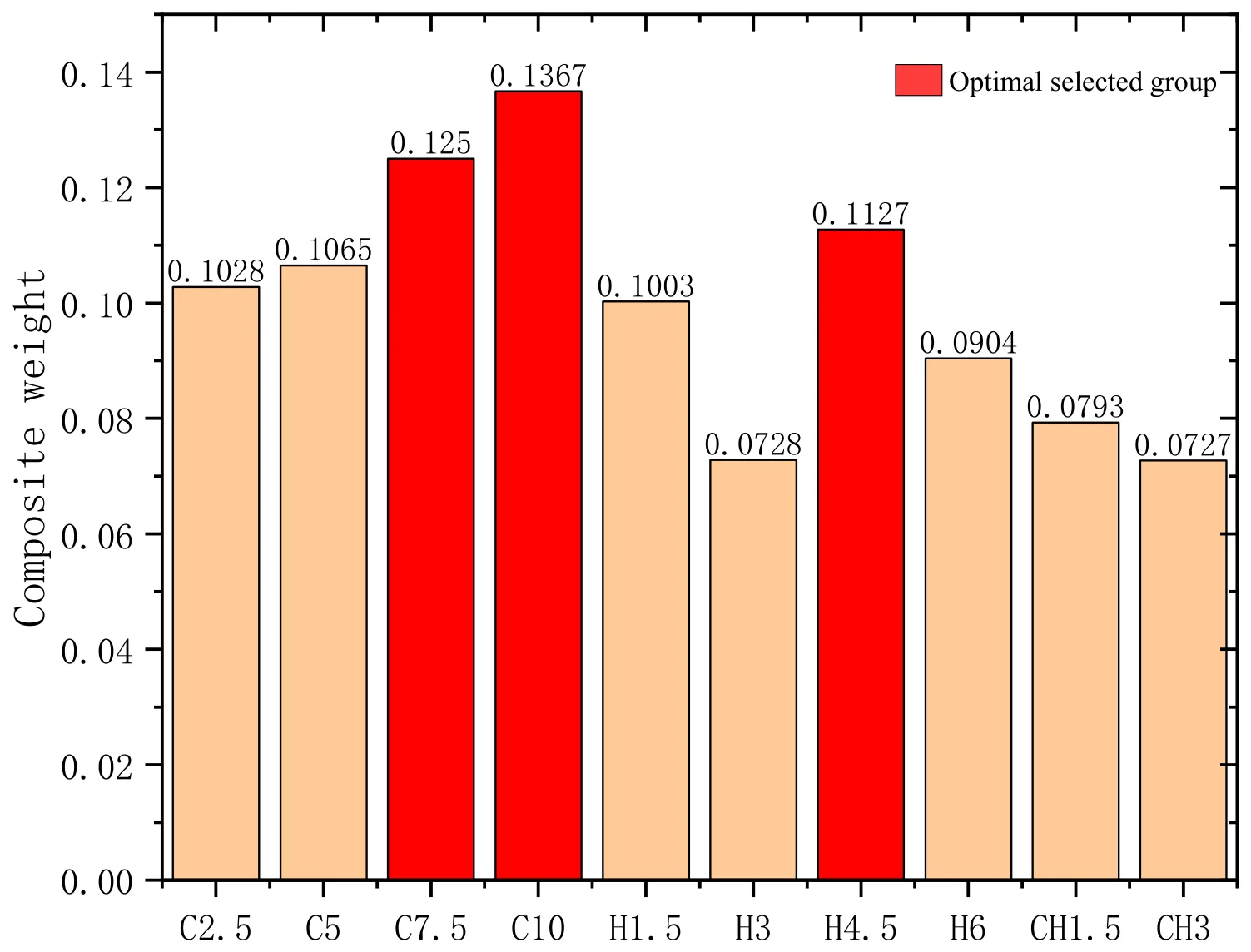
Evaluation results of analytic hierarchy process.

After the construction of the criterion layer and scheme layer judgment matrices, this study solved the eigenvectors of each evaluation indicator through YAAHP software and the arithmetic mean method. According to the calculations, the consistency indices and consistency ratios of the judgment matrices are approximately zero (e.g., CI = 4.9998 × 10^−5^ and CR = 5.6178 × 10^−5^ for the criterion layer matrix). This indicates that the pairwise comparison matrices exhibit near-complete mathematical consistency. This result is attributable to the ratio-based construction of the matrices from measured experimental data and confirms their internal logical coherence. Through the layer-by-layer synthesis of the weights of each indicator, the synthetic weights of each alternative scheme under the target of comprehensive performance optimization of radiation-shielding concrete are finally obtained. The weight values of each group are shown in Figure 8.

From the weight distribution, the comprehensive weights of the mixed groups (CH1.5 and CH3) are extremely low, both being lower than 0.1, indicating the poorest overall performance among all schemes. Further analysis suggests that the mixed groups experienced dual deterioration in both mechanical performance and construction applicability. On the one hand, the physical isolation effect of boron carbide powder and the strong chemical inhibition induced by borate ions exerts a negative synergistic effect within the system, leading to severe deterioration of the internal interfacial bonding performance of the material. On the other hand, the final setting times of the mixed groups reach to 37.73 h and 29.03 h, respectively, which substantially exceeds the tolerance limit for normal engineering construction. This pronounced risk of excessive retardation is assigned a high penalty weight in the evaluation system; consequently, despite their certain shielding efficiency, these mixtures failed to satisfy the requirements for comprehensive optimization.

The C10 and C7.5 groups rank as the top two alternatives in the comprehensive evaluation. Under the selected evaluation criteria and their assigned weights, the C10 group receives the highest overall score. This result is mainly associated with its relatively high boron content while maintaining acceptable setting time and mechanical performance among the evaluated mixtures.

It should be noted that no sensitivity analysis of the criterion weights was conducted in this study. Therefore, the ranking reflects the current evaluation framework, and changes in the relative weights of the criteria may affect the final ranking of the alternative mixtures.

The comprehensive weight of the H4.5 group remains at a moderately high level, ranking only below those of the C10 and C7.5 groups. This result indicates that the single-component boric acid admixture system can still exhibit certain comprehensive advantages when an appropriate dosage is adopted. Compared with the lower-dosage H1.5 and H3 groups, the H4.5 group contained a higher boron content, thereby providing a relative advantage in terms of shielding performance. However, because borate ions can significantly inhibit cement hydration, the final setting time and early-age mechanical strength development of the H4.5 group are still affected to some extent, which limits further improvement in its overall evaluation score. Overall, the H4.5 group demonstrates a relatively favorable balance within the single-component boric acid admixture system and may serve as an alternative option that accounts for both shielding efficiency and engineering applicability.

Ultimately, based on the ranking results of comprehensive performance weights, and considering the research needs for investigating the engineering applicability, indicator complementarity, and action mechanisms of different boron-containing material systems, the three groups with the highest comprehensive scores, namely C10, C7.5, and H4.5, are selected as the final optimized mix proportions.

Among them, C10 and C7.5 achieves the highest comprehensive scores within the boron carbide system, and can represent the mechanical properties, shielding potential, and construction performance of this system under relatively high boron loading conditions. As the highest-scoring group in the single-component boric acid admixture system, H4.5 can effectively reflect the comprehensive influence of modified boric acid on cement hydration, setting and hardening behavior, and mechanical properties under relatively high boric acid loading.

Although H4.5 is slightly inferior to C7.5 and C10 in terms of mechanical properties and setting time, boric acid is generally associated with retardation and strength deterioration. Given that H4.5 still exhibits a favorable strength recovery effect at a relatively high boric acid dosage, its selection can also be used to verify the effectiveness of modification treatment in weakening the adverse effects of boric acid and improving its engineering applicability. In addition, selecting H4.5, C7.5, and C10 as the optimized mix proportions enables a horizontal comparison between the boron carbide system and the modified boric acid system, thereby enhancing the scientific validity of subsequent microstructural analysis and neutron-shielding simulation verification.

In summary, this study finally determined C10, C7.5, and H4.5 as the optimized mix proportions, and selected them as the key objects for subsequent microscopic mechanism analysis and neutron-shielding performance simulation verification. It should be noted that because a sensitivity analysis of the criterion weights was not performed, the ranking should be interpreted within the present evaluation framework; variations in criterion weights may alter the relative ranking of the alternatives.

## 5. Analysis of Boron-Containing Concrete Component Based on XRD

**Figure 9:**
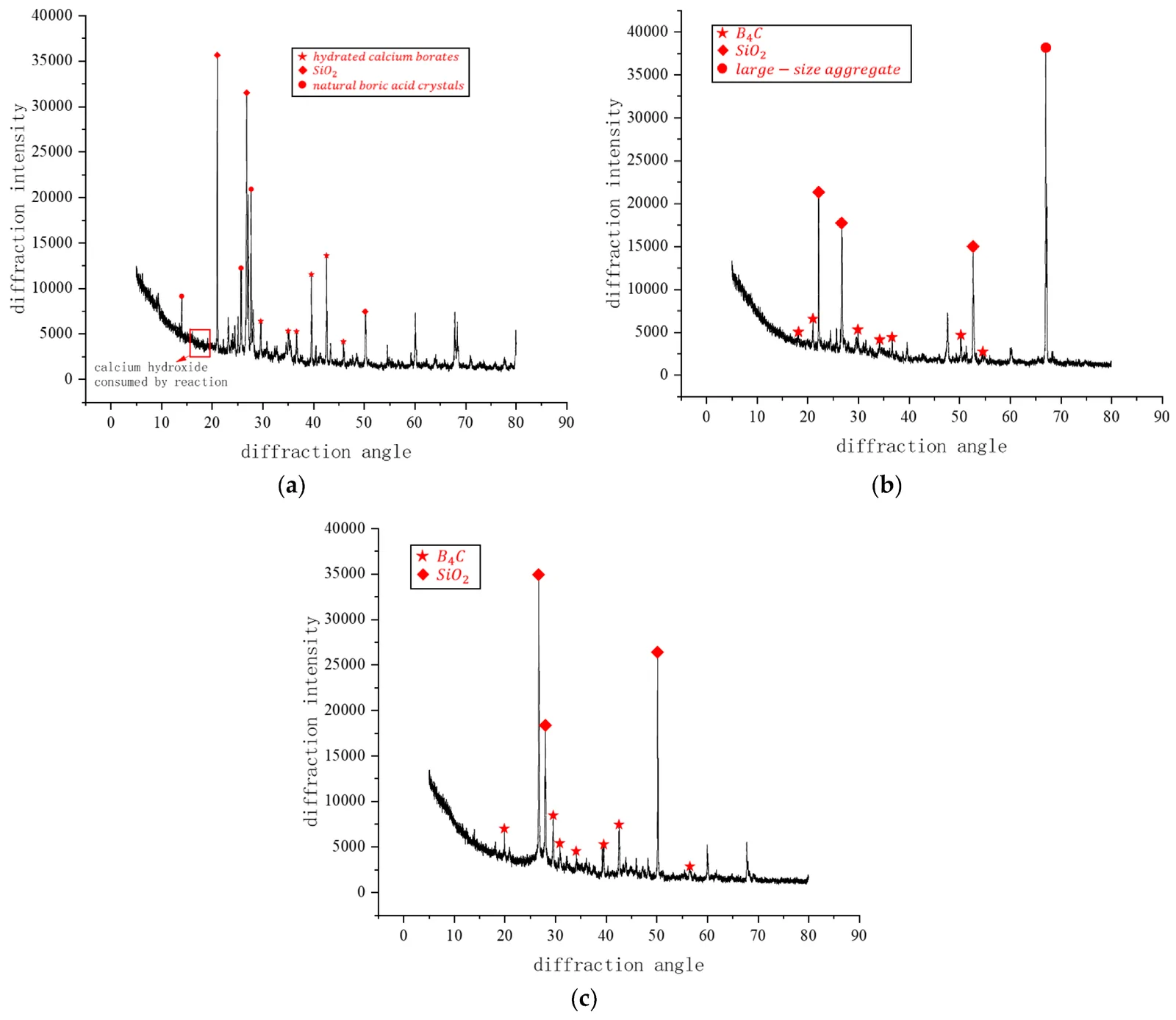
XRD analysis results: (**a**) H4.5; (**b**) C7.5; (**c**) C10.

The XRD analysis results are shown in Figure 9. As shown in Figure 9, the boron-containing phases in the H4.5 system mainly exist in two forms, hydrated calcium borate compounds and residual boric acid crystals. When the boric acid enters the alkaline pore solution of fresh concrete, it rapidly reacts with CH produced during hydration, forming crystalline hydrated calcium borate double salts with a general formula of xCaO·yB_2_O_3_·zH_2_O. These phases mainly include priceite, pandermite, calcium metaborate hexahydrate, calcium metaborate tetrahydrate, and colemanite, which exhibit pronounced diffraction peaks in the range of 29~46°. Meanwhile, owing to the relatively high dosage of boric acid, a portion of the unreacted boric acid recrystallizes within the pores as natural boric acid crystals, H_3_BO_3_, with characteristic peaks appearing at 14° and 27.5–28°. In addition, the diffraction peak of CH almost disappears, indicating that boric acid substantially consumes the free calcium sources in the cementitious system.

The boron in the C7.5 group concrete mainly exists jointly as the original B_4_C crystalline phase and a small amount of hydrated calcium borate phase generated by reaction. In XRD, the characteristic peaks of B_4_C are distributed in the range of 15~55°, indicating that boron carbide particles maintain relatively high chemical stability overall in the alkaline hydration environment of cement and mainly exist in the form of unreacted crystalline particles. At the same time, the surface oxide layer of industrial-grade B_4_C or B_2_O_3_ and free boron in impurities can be transformed into boric acid/borate after encountering water, and further react with Ca^2+^ and OH^−^ to form various hydrated calcium borate phases such as Ca(B(OH)_4_)_2_, Ca_2_B_6_O_11_·5H_2_O, CaB_6_O_10_·4H_2_O, and Ca_2_B_2_O_5_·H_2_O. Therefore, the boron-containing phases in C7.5 are dominated by stable B_4_C with reactive boron-containing hydration products as a supplement.

The types of boron-containing phases identified in the C10 group are generally consistent with those observed in C7.5. The phase assemblage is still dominated by the unreacted original B_4_C crystalline phase, accompanied by Ca–B–O–H-type hydrated calcium borate products formed through the transformation of surface boron oxides or boron-containing impurities [37]. Compared with C7.5, C10 contains a higher dosage of B_4_C. Therefore, the intensities of its characteristic diffraction peaks are stronger, whereas the peak positions remains essentially unchanged. This result indicates that increasing the B_4_C dosage mainly enriches the amount of crystalline B_4_C rather than altering its crystal structure. In this system, a small amount of reactive boron species can still form various hydrated calcium borate compounds in a strongly alkaline and calcium-rich environment. These XRD results suggest that B_4_C particles may remain largely inert under the tested conditions, whereas the surface oxide layer or boron-containing impurities may be possible sources of reactive boron species associated with the formation of boron-containing secondary phases. Further SEM/EDS and quantitative phase analyses are required to verify this interpretation.

It should be noted that, although this study comprehensively evaluated boron-containing concrete in terms of mechanical properties, setting time, boron content, and XRD characterization, several limitations remain: (1) Neutron transmission, attenuation, and irradiation tests were not conducted; therefore, the actual neutron-shielding performance cannot be experimentally verified. (2) Long-term irradiation, thermo-hygro coupled service conditions, and long-term durability were not evaluated. (3) XRD was used only for qualitative characterization, without SEM/EDS analysis, pore structure characterization, or quantitative phase analysis. Accordingly, the conclusions are limited to the materials and test conditions considered in this study.

## 6. Conclusions

In this study, high-abundance boron-10 boric acid and boron carbide were used as boron-containing functional components for concrete with potential neutron-shielding applications. A mix design framework integrating the Andreas and Andersen maximum packing density model, admixture regulation, pretreatment modification, AHP, and XRD characterization was established. This approach enables the quantitative optimization of boron-containing concrete by balancing mechanical performance, construction feasibility, and neutron-shielding potential. The main conclusions are as follows:

1. Boron carbide showed superior applicability in concrete compared with boric acid. The boron carbide mixtures exhibited shorter setting times, higher compressive and splitting tensile strengths, better performance stability, a more favorable effective ^10^B loading, and greater potential for neutron radiation shielding. For example, the C10 mixture still achieved a 28-day compressive strength of 47.3 MPa, whereas that of the H6 mixture was only 32.6 MPa. In contrast, within the dosage range investigated in this study, a negative interaction was observed between the two additives. The combined use of boric acid and boron carbide did not produce a synergistic effect, and its overall performance was inferior to that of systems incorporating a single boron source.
2. A practical dosage-selection strategy for neutron-shielding concrete was developed. By integrating boron loading, setting behavior, and mechanical performance, this study identified 7.5–10% boron carbide as the preferred dosage range and 4.5% modified boric acid as a feasible alternative. Among them, the C7.5 mixture achieved a 28-day compressive strength of 48.6 MPa, with an effective ^10^B content of 16.37‱. The C10 mixture achieved 47.3 MPa and 21.84‱, respectively. In contrast, the H4.5 mixture retained a compressive strength of only 51.3 MPa, with an effective ^10^B content of 10.44‱. This strategy overcomes the limitation of single-index evaluation and provides an engineering-oriented basis for optimizing boron-containing shielding concrete.
3. The distinct chemical activities of boron carbide and boric acid governed the phase evolution of the cement matrix. Boron carbide remained chemically inert and mainly acted as a physical micro-aggregate, contributing to pore refinement without disturbing C-S-H gel formation. Conversely, high-dosage boric acid showed strong chemical reactivity, consuming Ca (OH)_2_, forming crystalline hydrated calcium borate phases, and inhibiting the formation of normal hydration products such as ettringite and AFm.

## Figures and Tables

**Table 1:** Chemical composition of P.O. 52.5 ordinary Portland cement.

Compound	SiO_2_	CaO	Al_2_O_3_	MgO	SO_3_	Fe_2_O_3_	K_2_O	Others
**Content (%)**	23.886	54.826	8.226	4.424	3.097	3.734	0.704	1.103

**Table 2:** Chemical composition of boric acid.

Component	Chemical Formula	Content/Abundance
**Boric acid, enriched in ^10^B**	H_3_^10^BO_3_	≥99.9%
**Boron-10 isotope**	^10^B	96%
**Boron-11 isotope**	^11^B	approximately 4%
**Impurities**	—	≤0.1%

**Table 3:** Chemical composition of boron carbide.

Component	Content (%)	Component	Content (%)
B_4_C	97.18	Total C	19.81
Total B	78.94	Compound C	18.41
Compound B	78.77	Free C	1.40
Free B	0.17	Fe	0.16

**Table 4:** Mix proportions of boron-containing concrete (Unit: Kg/M^3^).

Group	Cement	Boric Acid	Boron Carbide	Fly Ash	Silica Fume	Sand	Small Stone	Large Stone	Water	Water Reducer	Early Strength Agent	Boron Element Content (‱)
KBZ	421.9	0.0	0.0	79.6	45.3	923.5	761.1	625.5	186.0	0.3828	0.0	0
C2.5	411.4	0.0	10.5	79.6	45.3	923.5	761.1	625.5	186.0	0.4438	0.0	5.46
C5	400.8	0.0	21.1	79.6	45.3	923.5	761.1	625.5	186.0	0.4921	0.0	10.92
C7.5	390.3	0.0	31.6	79.6	45.3	923.5	761.1	625.5	186.0	0.4921	0.0	16.37
C10	379.7	0.0	42.2	79.6	45.3	923.5	761.1	625.5	186.0	0.4921	0.0	21.84
H1.5	382.3	6.3	0.0	79.6	45.3	923.5	761.1	625.5	186.0	0.396	33.2	3.48
H3	376.5	12.7	0.0	79.6	45.3	923.5	761.1	625.5	186.0	0.46	32.7	6.96
H4.5	370.7	19.0	0.0	79.6	45.3	923.5	761.1	625.5	186.0	0.46	32.2	10.44
H6	364.9	25.3	0.0	79.6	45.3	923.5	761.1	625.5	186.0	0.46	31.7	13.92
CH1.5	370.7	6.3	12.7	79.6	45.3	923.5	761.1	625.5	186.0	0.428	0.0	10.23
CH3	370.7	12.7	6.3	79.6	45.3	923.5	761.1	625.5	186.0	0.428	32.2	10.03

**Table 5:** Criterion layer judgment matrix.

Dosage Optimization (A)	28 d Compressive Strength (B1)	28 d Splitting Tensile Strength (B2)	Final Setting Time (B3)	Boron Element Content (B4)
28 d compressive strength (B1)	1	4	1.8	1.2
28 d splitting tensile strength (B2)	0.25	1	0.45	0.3
Final setting time (B3)	0.556	2.222	1	0.667
Boron element content (B4)	0.833	3.333	1.5	1

**Table 6:** Details of expert participation in the AHP evaluation.

Information Category	Information Details
Number of experts	10
Main affiliations	Suzhou CGN Research Institute of Thermal Engineering; College of Civil Engineering and Architecture, Zhejiang University
Professional titles	Senior professional titles, associate senior professional titles, professors, associate professors, and technical professionals with extensive engineering experience
Nuclear engineering-related fields	Nuclear power engineering, nuclear facility materials, radiation-shielding materials, and performance evaluation of nuclear engineering materials
Civil engineering materials-related fields	Cement-based composites, concrete materials science, structural engineering materials, and concrete durability
Scope of AHP participation	Pairwise comparisons to determine the weights of criteria-level indicators; no participation in subjective scoring of alternative schemes

**Table 7:** Judgment matrix of alternative schemes under the 28-day compressive strength criterion.

Compressive Strength (B_2_)	C2.5 (C_1_)	C5 (C_2_)	C7 (C_3_)	C10 (C_4_)	H1.5 (C_5_)	H4.5 (C_6_)	CH1.5 (C_7_)	CH3 (C_8_)
C2.5 (C_1_)	1	1.059	1.142	1.173	1.161	1.082	1.35	1.729
C5 (C_2_)	0.944	1	1.078	1.108	1.096	1.021	1.275	1.632
C7 (C_3_)	0.876	0.928	1	1.028	1.017	0.947	1.183	1.514
C10 (C_4_)	0.852	0.903	0.973	1	0.99	0.922	1.151	1.474
H1.5 (C_5_)	0.861	0.912	0.984	1.011	1	0.932	1.163	1.489
H4.5 (C_6_)	0.924	0.979	1.056	1.085	1.073	1	1.248	1.598
CH1.5 (C_7_)	0.741	0.784	0.846	0.869	0.86	0.801	1	1.28
CH3 (C_8_)	0.578	0.613	0.661	0.679	0.672	0.626	0.781	1

**Table 8:** Judgment matrix of alternative schemes under the 28-day splitting tensile strength criterion.

Splitting Tensile Strength (B_2_)	C2.5 (C_1_)	C5 (C_2_)	C7 (C_3_)	C10 (C_4_)	H1.5 (C_5_)	H4.5 (C_6_)	CH1.5 (C_7_)	CH3 (C_8_)
C2.5 (C_1_)	1	1.047	1.094	1.216	1.089	0.985	1.422	1.552
C5 (C_2_)	0.955	1	1.045	1.162	1.04	0.94	1.358	1.482
C7 (C_3_)	0.914	0.957	1	1.112	0.996	0.9	1.3	1.418
C10 (C_4_)	0.822	0.861	0.9	1	0.896	0.81	1.169	1.276
H1.5 (C_5_)	0.918	0.961	1.004	1.116	1	0.904	1.306	1.424
H4.5 (C_6_)	1.016	1.063	1.111	1.235	1.106	1	1.444	1.576
CH1.5 (C_7_)	0.703	0.736	0.769	0.855	0.766	0.692	1	1.091
CH3 (C_8_)	0.645	0.675	0.705	0.784	0.702	0.635	0.917	1

**Table 9:** Judgment matrix of alternative schemes under the final setting time criterion.

Final Setting Time (B_3_)	C2.5 (C_1_)	C5 (C_2_)	C7 (C_3_)	C10 (C_4_)	H1.5 (C_5_)	H4.5 (C_6_)	CH1.5 (C_7_)	CH3 (C_8_)
C2.5 (C_1_)	1	1.101	1.092	1.136	0.731	1.339	4	3.078
C5 (C_2_)	0.909	1	0.992	1.032	0.665	1.217	3.634	2.796
C7 (C_3_)	0.916	1.008	1	1.04	0.67	1.227	3.663	2.819
C10 (C_4_)	0.88	0.969	0.961	1	0.644	1.179	3.521	2.709
H1.5 (C_5_)	1.367	1.505	1.493	1.553	1	1.831	5.469	4.208
H4.5 (C_6_)	0.747	0.822	0.815	0.848	0.546	1	2.987	2.298
CH1.5 (C_7_)	0.25	0.275	0.273	0.284	0.183	0.335	1	0.769
CH3 (C_8_)	0.325	0.358	0.355	0.369	0.238	0.435	1.3	1

**Table 10:** Judgment matrix of alternative schemes under the boron content criterion.

Boron Content (B_4_)	C2.5 (C_1_)	C5 (C_2_)	C7 (C_3_)	C10 (C_4_)	H1.5 (C_5_)	H4.5 (C_6_)	CH1.5 (C_7_)	CH3 (C_8_)
C2.5 (C_1_)	1	0.5	0.334	0.25	1.569	0.523	0.534	0.544
C5 (C_2_)	2	1	0.667	0.5	3.138	1.046	1.067	1.089
C7 (C_3_)	2.998	1.499	1	0.75	4.704	1.568	1.6	1.632
C10 (C_4_)	4	2	1.334	1	6.276	2.092	2.135	2.177
H1.5 (C_5_)	0.637	0.319	0.213	0.159	1	0.333	0.34	0.347
H4.5 (C_6_)	1.912	0.956	0.638	0.478	3	1	1.021	1.041
CH1.5 (C_7_)	1.874	0.937	0.625	0.468	2.94	0.98	1	1.02
CH3 (C_8_)	1.837	0.918	0.613	0.459	2.882	0.961	0.98	1

**Table 11:** Average random consistency index [32].

Matrix Order n	1	2	3	4	5	6	7	8
RI	0	0	0.52	0.89	1.12	1.24	1.36	1.48

**Table 12:** Parameters, equations, and numerical values involved in the AHP.

Parameters	Equations	Values
W	$W={\left(w_{1},w_{2},\cdots ,w_{n}\right)}^{T}$	${\left(0.387,\;0.097,\;0.323,\;0.194\right)}^{T}$
$\lambda_{\max}$	$\lambda_{\max}=\frac{1}{n}\overset{n}{\underset{i=1}{\sum }}\frac{{\left(\mathrm{AW}\right)}_{i}}{w_{i}}$	4.00015
P	$P=\overset{n}{\underset{k=1}{\sum }}w_{k}p_{k}$	(0.1028, 0.1065, 0.1250, 0.1367, 0.1003, 0.0728, 0.1127, 0.0904, 0.0793, 0.0727)^T^
CI	$\mathrm{CI}=\frac{\lambda_{\max}-n}{n-1}$	4.9998115106 × 10^−5^
RI	as shown in Table 11	0.89
CR	$\mathrm{CR}=\frac{\mathrm{CI}}{\mathrm{RI}}$	5.6177657422 × 10^−5^

## Data Availability

The original contributions presented in this study are included in the article. Further inquiries can be directed to the corresponding authors.
